# Baseline PD-L1 expression on circulating immune cells as a predictor of survival and immune-related adverse events in extensive-stage small-cell lung cancer patients treated with durvalumab and carboplatin-etoposide (NCT04712903 Trial)

**DOI:** 10.1186/s12967-026-07896-7

**Published:** 2026-02-28

**Authors:** Aida Piedra, Albert Guinart-Cuadra, Sergio Martínez-Recio, Maria Mulet, Carlos Zamora, Rubén Osuna-Gómez, Elisabet Cantó, Maria Angels Ortiz, José Alejandre, Andrés Barba, Judit Sanz-Beltrán, Jorgina Serra-López, Dolores Isla, Edurne Arriola, Luis Paz-Ares, María Pilar Diz-Taín, Alberto Luis Moreno, Ángel Callejo, Silvia Vidal, Margarita Majem

**Affiliations:** 1 Medical Oncology Department, Hospital de la Santa Creu i Sant Pau, Sant Quintí 89, Barcelona, 08025 Spain; 2 Department of Medicine, Universitat Autònoma de Barcelona (UAB), Barcelona, Spain; 3 Inflammatory Diseases, Institut de Recerca Sant Pau (IR SANT PAU), Sant Qunití 77-79, Barcelona, 08041 Spain; 4 Department of Cell Biology, Physiology and Immunology, Autonomous University of Barcelona, Bellaterra, Spain; 5 Centre for Biomedical Research in Liver and Digestive Diseases Network (CIBEREHD), Madrid, Spain; 6 Medical Oncology Department, Hospital Universitario Lozano Blesa, Zaragoza, Spain; 7 Medical Oncology Department, Hospital del Mar – CIBERONC, Barcelona, Spain; 8 Medical Oncology Department, Hospital Universitario 12 Octubre, Madrid, Spain; 9 Medical Oncology Department, Complejo Asistencial Universitario de León, León, Spain; 10 Medical Oncology Department, Hospital Universitario Reina Sofía, Córdoba, Spain; 11 AstraZeneca Farmacéutica Spain, Madrid, Spain

**Keywords:** Predictive biomarker, Extensive stage small-cell lung cancer (ES-SCLC), Circulating immune cells, Circulating PD-L1^+^ monocytes, Circulating PD-L1^+^ neutrophils, Immunotherapy

## Abstract

**Introduction:**

Despite improved efficacy with first-line immune checkpoint inhibitors plus platinum-based chemotherapy for extensive-stage small cell lung cancer (ES-SCLC), long-term survival remains limited. There is currently no available predictive biomarker to identify which patients would benefit most from this treatment. We hypothesized that pre-treatment PD-L1 expression on circulating immune cells might predict survival outcomes and toxicity.

**Material and methods:**

This prospective, multi-center observational study included patients with untreated ES-SCLC treated with first-line durvalumab plus platinum-based chemotherapy. The percentages of circulating PD-L1^+^ immune cells at baseline were analysed by flow cytometry to assess their association with survival outcomes and the development of immune-related adverse events (irAEs).

**Results:**

Among 41 patients with ES-SCLC, 65.9% were male, 73.2% had an ECOG-PS 1, 9.8% had central nervous system (CNS) metastases and 31.7% had liver metastases. Sixteen patients (39%) experienced irAEs. Median PFS was longer in patients with high percentages of circulating PD-L1^+^ monocytes compared to those with low percentages: 8.97 months (95% CI NR to NR) vs. 5.97 months (95% CI 4.65 to 7.28), *p* = 0.007. There was a trend toward longer median OS in patients with ES-SCLC and high percentages of circulating PD-L1^+^ monocytes versus low percentages: NR (95% CI NR-NR) vs. 9.13 months (95% CI 6.34 to 11.92), *p* = 0.092. Patients with higher circulating PD-L1^+^ neutrophils correlated with the development of irAES (*p* = 0.007).

**Conclusions:**

Our results showed a statistically significant longer PFS in patients with ES-SCLC and high percentages of circulating PD-L1^+^ monocytes. This suggests PD-L1 expression on monocytes might be established as a predictive biomarker for patients with ES-SCLC treated with upfront chemo-immunotherapy.

**Trial registration:**

NCT04712903 Trial. Last registered 1 December 2025, https://www.clinicaltrials.gov/study/NCT04712903.

**Supplementary Information:**

The online version contains supplementary material available at 10.1186/s12967-026-07896-7.

## Introduction

Small cell lung cancer (SCLC) represents a highly aggressive subtype of lung cancer, with more than 60% of patients diagnosed at extensive-stage (ES) [1]. Treatment with anti programmed death-(ligand) 1 [PD-(L)1] in combination with platinum and etoposide was established as the standard of care, based on the phase III clinical CASPIAN and IMpower133 trials [2–4]. Prognosis remains poor even after their approval, reporting a median progression-free survival (PFS) and overall survival (OS) of approximately 5 months and 13 months, respectively.

To date, significant efforts have been made to identify predictive biomarkers for survival in ES-SCLC patients treated with first-line immune check-point inhibitors (ICIs) with limited conclusions. High tumor mutational burden (TMB) has been proposed as a potential predictor of benefit [5], although results remain inconclusive. Retrospective analyses have associated specific metastatic sites – particularly brain and liver metastasis- with poorer outcomes [6–9]. Human leukocyte antigen (HLA) has also been proposed to have an influence on outcomes in SCLC, as Zhao et al. reported the positive effect of the HLA-B44 supertype on PFS, thus suggesting the prognostic value of HLA-B44 in SCLC, reporting an enhanced cytolytic activity and an increased expression of immune-related genes, including MHC-II, interferon-gamma, and chemokine signatures, although further validation is warranted [10]. Mayor Histocompatibility Complex (MHC) expression [11], or transcriptome profiles [12] have also emerged as potential relevant factors, where mRNA expression in the inflammed-SCLC (SCLC-I) subtype has been reported as a potential benefit [13]. However, transcriptome analysis implies long turnaround times and limited availabilty accross sites which impairs its feasibility.

Among all, PD-L1 expression has been the most extensively investigated. Although PD-L1 expression in tumor cells correlates with ICI efficacy in several malignancies, including non-small cell lung cancer (NSCLC) [14, 15], to our knowledge, its predictive value in first-line ES-SCLC remains unconfirmed [6, 16]. Consequently no validated biomarker is recommended to guide personalized treatment decisions in this setting,

underscoring the need for alternative sources of predictive information.

Previous studies in NSCLC and other tumors [17] have explored other sources of PD-L1 expression on circulating components as potential biomarkers, including circulating tumor cells [18–21], leukocyte-platelet complexes [22–24], platelets (PLTs) [25], tumor-associated cells [26] and circulating immune cells [27–29]. Circulation biomarkers offer a key advantage: they are accessible through peripheral blood tests and enable longitudinal monitoring. Our previous research demonstrated that NSCLC patients with high percentages of PD-L1^+^ monocytes, neutrophils, platelets and PD-L1^+^ platelet microparticles exhibited a prolonged PFS. Furthermore, high PD-L1^+^ monocyte levels correlated with longer OS, compared to healthy donors (HD) [29]. In ES-SCLC patients, exploratory analyses on PD-L1 expression revealed consistent survival benefits across the different expression subgroups [4] and, to our knowledge, PD-L1^+^ circulating immune cells have not yet been studied in ES-SCLC patients.

Regarding toxicity, anti PD-(L)1 blockade agents can induce inflammatory side effects in up to 50% of patients, known as *immune-related adverse events* (irAEs) [30]. In some studies, irAEs have correlated with improved survival [31–33]. Because their onset and potential complications are largely unpredictable, early recognition is essential [30]. Promising predictive biomarkers for irAEs have been proposed, including high prognostic nutritional index at baseline [34] and circulating leukocyte-platelet complexes in advanced NSCLC [35]. Of note, the combination of percentages of CD4^+^PLT^+^ and CD14^+^PLT^+^ were associated with higher risk and severity of irAEs [35]. However, the relationship between PD-L1^+^ circulating immune cells and irAE development has not been explored in NSCLC or SCLC.

In the absence of an established predictive biomarker for ES-SCLC patients receiving first-line durvalumab plus platinum-etoposide, and given previous evidence linking PD-L1 expression on monocytes with clinical outcomes in NSCLC, we hypothesized that PD-L1 expression on circulating immune cells might influence both survival and toxicity outcomes, helping us stratifying patients which could benefit most from this treatment. Our objectives were, firstly, to determine baseline levels of PD-L1 expression on circulating immune cells before treatment, and secondly, to evaluate their association with survival and toxicity, with the aim of identifying a novel, non-invasive prognostic and/or predictive biomarker for first-line ES-SCLC.

## Materials and methods

### Patient population

This study was conducted as a sub-analysis of the multicenter phase IIIb CANTÁBRICO trial (NCT04712903 Trial, EudraCT: 2020-002328-35) [36]. Elegible patients were adult patients with an ECOG performance status 0–1 and measurable histologically or cytologically confirmed ES-SCLC, either treatment-naive or relapsed after ≥ 6 months of limited-stage SCLC, without previous exposure to immunotherapy. All patients received first-line durvalumab and platinum-etoposide. Asymptomatic, untreated or previously treated brain metastases were allowed.

Patients were enrolled between February 2021 and May 2021, and data cut-off was January 2024. Blood samples were collected before starting treatment, as per protocol: 14 days maximum before day 1 (pre-dose) and were centrally analyzed at Institut Institut de Recerca in Hospital de la Santa Creu i Sant Pau (Barcelona, Spain) where, additionally, 22 Healthy Donors (HD), voluntarily provided blood samples.

Treatment discontinuation creteria included disease progression (per Response Evaluation Criteria in Solid Tumors (RECIST) version 1.1 [37]) by investigator, consent withdrawal or unacceptable toxicity. Radiologic assessments were performed at baseline and periodically thereafter, following investigator´s discretion. Clinical symptoms, physical examinations and laboratory test were collected at baseline and before each treatment cycle according to the study protocol. Data were anonymized upon inclusion in a database and collected from central electronic records. Written informed consent was obtained from all participants, and the study was approved by the local Ethics Committee and collaborating institutions.

### Immune-related adverse events (irAEs)

irAEs were defined as an adverse event of immunologic origin related to durvalumab, in accordance with the study protocol, and graded using the NCI CTCAE v5.0 [38]. When analysing the correlation between severity of irAEs and the percentage of circulating PD-L1^+^ immune cells, only the most severe irAE per patient was considered. For overall incidence analyses, all irAEs were included.

Because irAEs are time-dependent, a 12-month time-point was used to assess their occurrence; patients who discontinued treatment before that time for any reason were excluded from these analyses.

### Sample collection and blood staining for flow cytometry

Whole blood samples were collected at Institut de Recerca in Hospital de la Santa Creu i Sant Pau in heparinized BD Vacutainer tubes (BD, Franklin Lakes, NJ) before starting treatment. A 100ul aliquot of TransFix-treated (a cellular antigen stabilizing reagent) whole blood samples was incubated with anti-CD3-Viogreen, anti-CD8-Vioblue, anti-CD20-APCVio770, anti-CD16-PerCPVio700 (all from Miltenyi Biotec, Bergisch Gladbach, Germany), anti-CD41a-FITC (Immunotools, Friesoythe, Germany), anti-CD14-APC (BD) and anti-PD-L1-PE (BioLegend, San Diego, USA) monoclonal antibodies and their corresponding isotype controls. After incubation, red blood cells were lysed and white cells were fixed using BD FACS lysing solution (BD), washed with 2 mL of PBS 1X and resuspended in 400 µL of PBS 1X for flow cytometry analysis. BD FACS Lysing Solution was used with short incubation (≤ 10 min, room temperature and protected from light) so that PE-Cy7 remained stable.

### Flow cytometry analyses of PD-L1 on different cell populations

PD-L1 expression was analyzed across distinct immune subsets using Forward scatter (FSC) and Side scatter (SCC) parameters. Lymphocytes were gated based on size and complexity (FSC/SSC) and further characterized by surface markers. CD8⁺ T lymphocytes were identified as CD3⁺CD8⁺ cells, and CD4⁺ T lymphocytes were approximated as CD3⁺CD8⁻ cells, given that CD4 was not included in the staining panel. B cells were defined as CD3⁻CD20⁺. NK cells were operationally identified as CD3⁻CD8⁺CD16⁺, a well-described subset encompassing the majority of circulating cytotoxic NK cells. We acknowledge that CD16⁻CD56⁺ NK cell subsets could not be separately evaluated due to the absence of CD56 in the panel. Monocytes were gated based on CD14 expression (CD14^+^) while neutrophils were gated as SSC-high, CD14-CD16^+^ cells. To analyze PLTs in blood after centrifugation, samples were acquired using FSC and SSC on a logarithmic scale. Blood PLTs were identified as CD41a^+^ events > 1 μm, using predefined gating regions. Samples were acquired using a MACSQuant Analyzer 10 flow cytometer (Miltenyi Biotec). The percentage of PD-L1 positive cells (PD-L1+) according to FMO controls and event/µL for each population was obtained using FlowJo version X (FlowJo LLC, Ashland, USA).

### Statistical analyses

Descriptive statistics summarized baseline variables. Qualitative variables were expressed as counts and percentages, and quantitative variables as medians (interquartile ranges) or means ± standard error (s.e.m.) where appropriate.

The Kolmogorov–Smirnov test assessed normality. Parametric comparisons employed Student’s t-test or ANOVA, while non-parametric comparisons used Mann–Whitney or Kruskal–Wallis tests. Fisher’s exact and χ² tests were used for categorical variables.

Correlations were tested with Pearson or Spearman coefficients, depending on data distribution.

To establish categorical “low” and “high” PD-L1 expression groups, we calculated 95% confidence interval (CI) cut-offs using HD values. Each patient’s percentage of PD-L1⁺ CD4⁺/CD8⁺ T cells, CD20⁺ B cells, NK cells, neutrophils, monocytes, and platelets was classified accordingly.

Progression-free survival (PFS) was defined from treatment initiation to disease progression or last follow-up without progression. Overall survival (OS) was defined from treatment initiation to death or last follow-up if alive.

Survival analyses used Kaplan–Meier estimation and log-rank comparisons. Cox regression was applied for multivariate analysis under proportional-hazards assumptions. Statistical significance was set at *p* < 0.05 (two-sided). Analyses were performed with GraphPad Prism v8 and IBM SPSS Statistics v25; figures were created with BioRender.

## Results

### Patient characteristics and survival outcomes

In this sub-study of the CANTABRICO trial [36], pretreatment samples were collected from 41 patients. Clinicopathological characteristics are summarized in Table 1. The median age was 64 years (range: 48–84), 65.9% were male and 51.2% were current smokers. Eastern Cooperative Oncology Group performance status (ECOG-PS) was 1 in 30 patients (73.2%). At inclusion, 4 patients (9.8%) had central nervous system (CNS) metastases, 13 patients (31.7%) had bone metastases and 13 patients (31.7%) had liver metastases. A total of 37 (90.2%) completed the planned four cycles of chemotherapy and durvalumab. The main reasons for treatment discontinuation were disease progression (24 patients, 58.5%) and death (7 patients, 17.1%), with. disease progression being the most common cause of death(80%). At the time of data cut-off, 11 patients (26.8%) were alive. With a median follow-up of 10.19 months (95% CI 8.33–12.06, IQR 6.74–21.03), the median OS was 10.3 months (95% CI 8.7–11.8) and the median PFS was 7.0 months (95% CI 6.0-8.1).

**Table 1:** Patient characteristics

Category – *N* (%)	Total (*N* = 41)
**Sex**	
Male	27 (65.9)
Female	14 (34.1)
**Race**	
White	37 (90.2)
Others	4 (9.8)
**Age – years**	
Median	64
Range	48–84
**ECOG-PS**	
0	11 (26.8)
1	30 (73.2)
**Smoking habits**	
Former smoker	20 (48.8)
Current smoker	21 (51.2)
**BMI (kg/m2)**	
< 18,5	1 (2.4)
18,5–24,9	13 (31.7)
≥ 25	27 (65.9)
**CNS metastases**	
No	37 (90.2)
Yes	4 (9.8)
**Bone metastases**	
No	28 (68.3)
Yes	13 (31.7)
**Liver metastases**	
No	28 (68.3)
Yes	13 (31.7)

ECOG-PS: Eastern Cooperative Oncology Group performance status; light-smoker: less than 100 cigarettes/life; BMI: body mass index, CNS: central nervous system

### High percentages of baseline PD-L1^+^ circulating immune cells in ES-SCLC

**Fig. 1:**
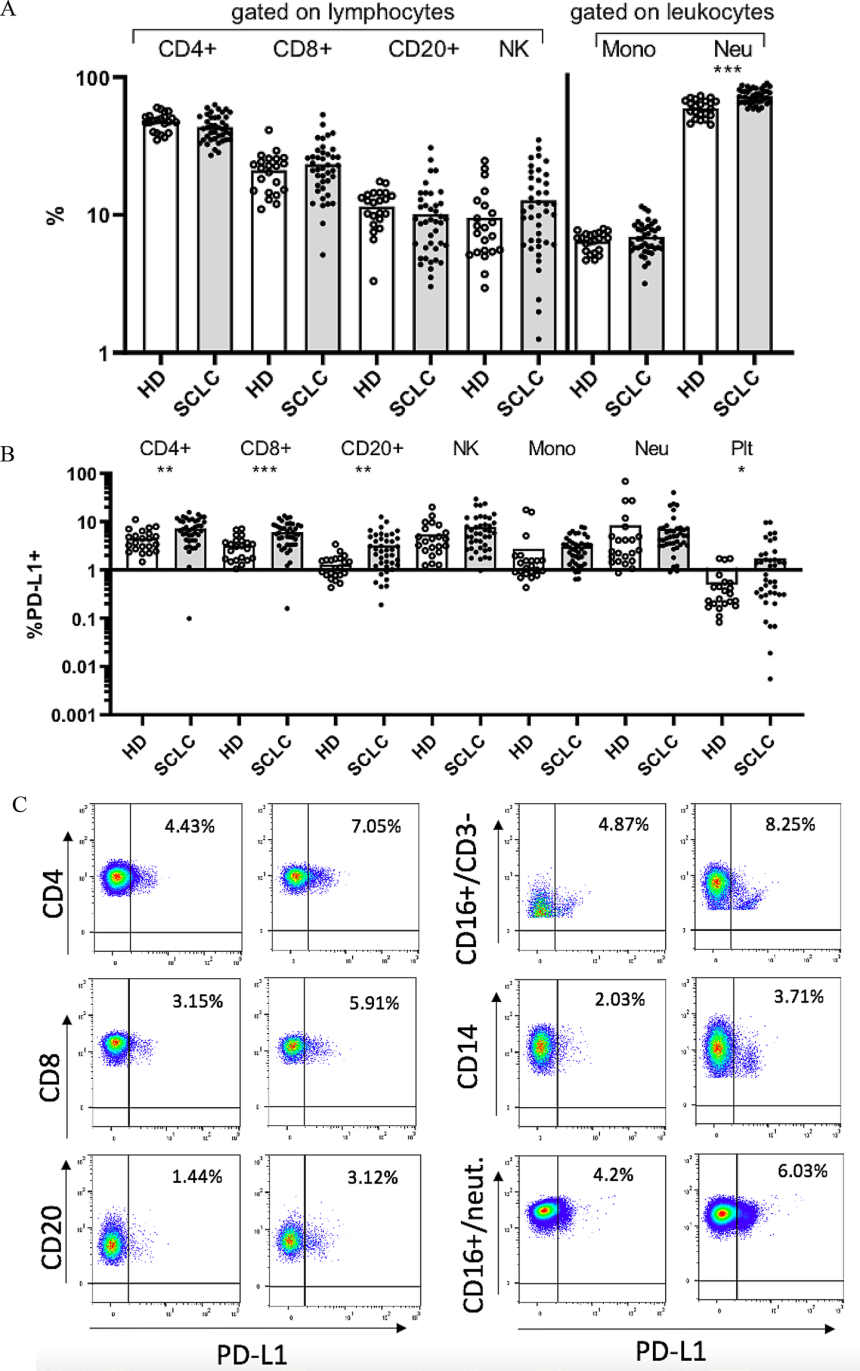
Baseline circulating cells in ES-SCLC patients. **A**: Percentages of baseline circulating cells in ES-SCLC patients (*N* = 41) (SCLC) compared to healthy donors (*N* = 22) (HD), represented by dot plots. **B**: Percentages of baseline circulating PD-L1^+^ circulating cells in ES-SCLC patients compared to HD, represented by dot plots. **C**: Representative flow cytometry images of PD-L1 expression (PD-L1^+^) on circulating cells in ES-SCLC patients and HD. *PD-L1: programmed death-ligand 1*, *HD*: healthy donor, *SCLC*: small-cell lung cancer, *NK*: natural killer cells, *Mono*: monocytes, *CD14*: monocytes, *Neu*: neutrophils, *PLTs*: platelets. * *p* < 0.05, **p < 0.01 and ***p < 0.001

Neutrophils, CD4^+^ T lymphocytes and CD8^+^ T lymphocytes were the predominant subpopulations in pre-treatment samples. Compared to HD, ES-SCLC patients exhibited higher percentages of neutrophils (*p* < 0.001) (Fig. 1A), PDL1^+^ CD4^+^ T lymphocytes (*p* < 0.01), PDL1^+^ CD8^+^ T lymphocytes (*p* < 0.001), PDL1^+^ CD20^+^ B lymphocytes (*p* < 0.01) and PDL1^+^ platelets (*p* < 0.05).Trends toward higher percentages of PD-L1^+^ NK cells, PD-L1^+^ neutrophils and PD-L1^+^ monocytes were also observed (Fig. 1B-C).

### High percentages of baseline PD-L1^+^ circulating monocytes were associated with better outcomes

**Fig. 2:**
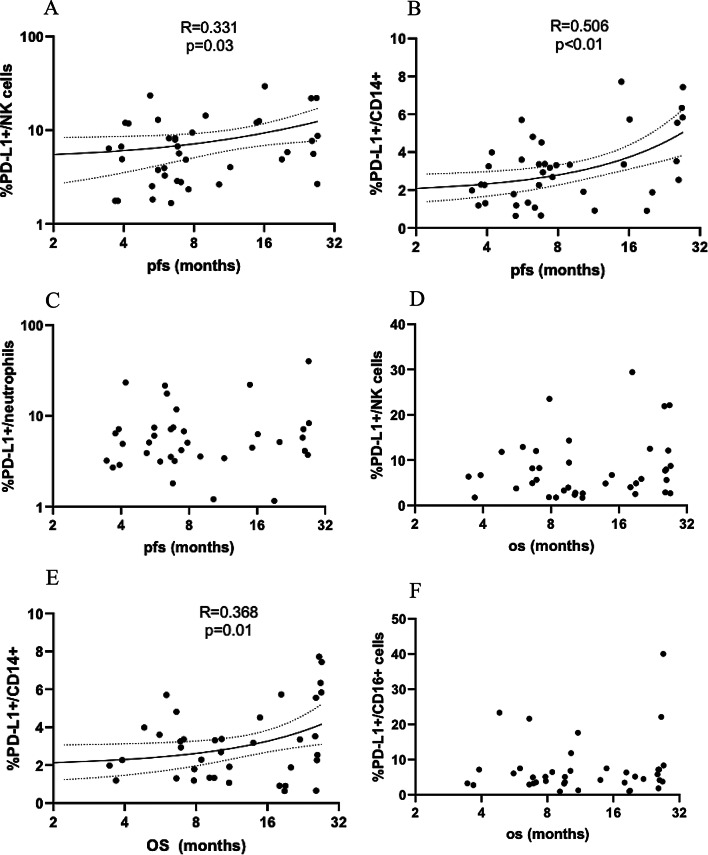
Correlation between pretreatment percentages of PD-L1^+^ and survival outcomes (Spearman correlation (R)) in patients with ES-SCLC treated with durvalumab-platinum-etoposide. **A**: PFS and PD-L1^+^ NK cells **B**: PFS and PD-L1^+^ monocytes. **C**: PFS and PD-L1^+^ neutrophils. **D**: OS and PD-L1^+^ NK cells. **E**: OS and PD-L1^+^ monocytes. **F**: OS and PD-L1^+^ neutrophils. *OS*: overall survival; *PFS*: progression-free survival; *OS*: overall survival

Percentages of PD-L1^+^ NK cells (*R* = 0.331, *p* = 0.03) (Fig. 2A) and PD-L1^+^ monocytes (*R* = 0.506, *p* < 0.01) (Fig. 2B) correlated with PFS. No significant correlation was observed for the percentage of PD-L1^+^ neutrophils and PFS (Fig. 2C). As innate immune system cells (NK cells, monocytes, neutrophils) statistically correlated with PFS, our results will focus on these subpopulations. Neither the percentages of PD-L1^+^ T nor B lymphocytes significantly impacted on PFS (*Supplementary Fig. 1A-C*).

In terms of OS, the percentage of PD-L1^+^ monocytes correlated with OS (*R* = 0.368, *p* = 0.01) (Fig. 2E), while other immune subpopulation showed no significant correlation (Supplementary Fig. 1D-F).

Based on Spearman correlations, patients were categorized into low/high PD-L1^+^ groups (low PD-L1^+^ circulating cells and high PD-L1^+^ circulating cells) using 95% CI-derived cut-offs from HD values: CD4^+^ T cells 9.19%, CD8^+^ T cells 6.64%, CD20^+^ B cells 2.70%, NK cells 11.20%, neutrophils 16.41%, monocytes 3.30% (Supplementary Table 1). Patient´s characteristics were well balanced between groups: no differences were found in terms of sex, BMI, ECOG-PS, the presence of CNS metastases, bone metastases, liver metastases or age (Supplementary Table 2).

**Fig. 3:**
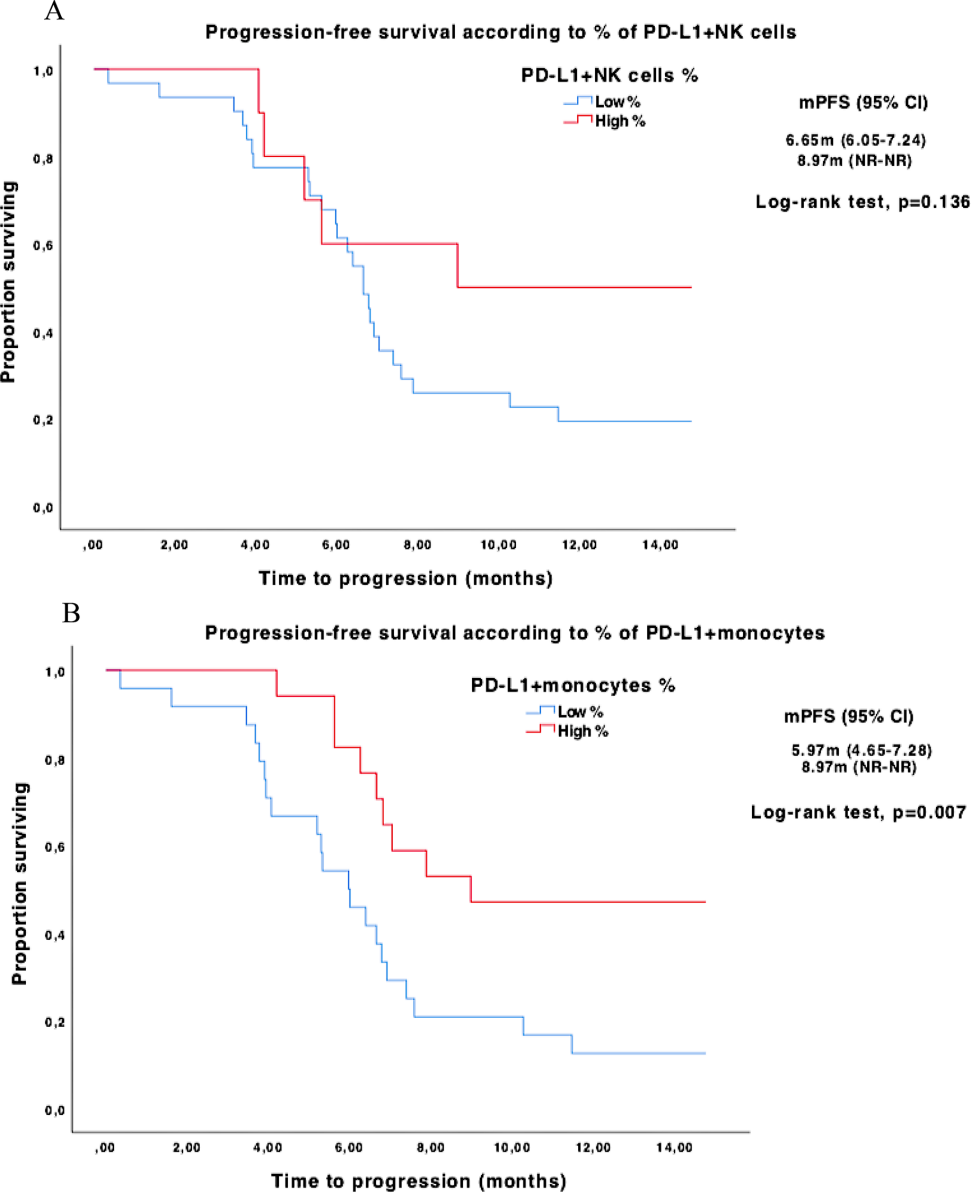

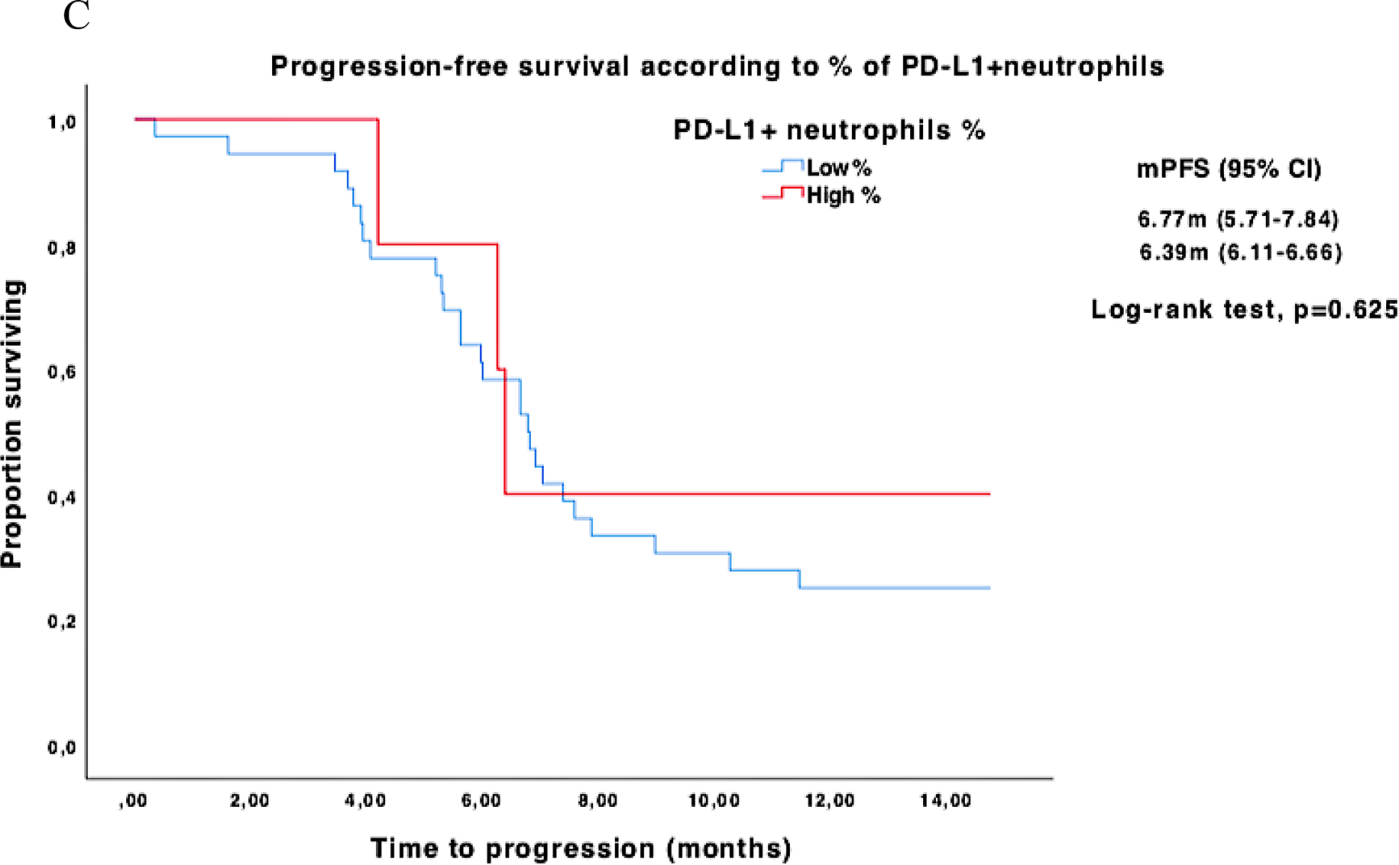
Kaplan-Meier curves for PFS according to low and high percentages of PD-L1^+^ circulating immune cells. **A**: PD-L1^+^NK cells. **B**: PD-L1^+^monocytes. **C**: PD-L1^+^neutrophils

Patients with high PD-L1^+^ monocyte percentages had significantly longer median PFS compared to low-percentage patients: 8.97 (95% CI NR to NR) vs. 5.97 months (95% CI 4.65 to 7.28), *p* = 0.007 (Fig. 3B).

Trend toward longer PFS were observed in high percentages of PD-L1^+^ NK cells [median PFS 8.97 (95% CI NR to NR) vs. 6.65 months (95% CI 6.05 to 7.24), *p* = 0.136 (Fig. 3A)] and high percentages of PD-L1^+^ B lymphocytes [median PFS 7.39 (95% CI 6.01 to 8.76) vs. 5.61 months (95% CI 4.80 to 6.43), *p* = 0.065 (Supplementary Fig. 2C)]. No differences in median PFS were observed in PD-L1^+^ neutrophils (Fig. 3C), nor PD-L1^+^ T lymphocytes or PD-L1^+^ platelets (Supplementary Fig. 2A–B).

Notably, median PFS was significantly longer in patients with high percentages of PD-L1^+^ monocytes without CNS metastases [8.97 months (95% CI NR to NR) vs. 5.32 months (95% CI 3.95 to 6.69), *p* = 0.008 (Supplementary Fig. 2E)]; without bone metastases [NR (95% CI NR to NR) vs. 6.90 months (95% CI 5.97 to 7.84), *p* = 0.038 (Supplementary Fig. 2F)]; without liver metastases [NR (95% CI NR to NR) vs. 6.65 months (95% CI 5.60 to 7.69), *p* = 0.039 (Supplementary Fig. 2G)] and ECOG-PS 1 [8.97 months (95% CI NR to NR) vs. 5.97 months (95% CI 5.01 to 6.92), *p* = 0.006 (Supplementary Fig. 2H)].

OS trends mirrored PFS but did not reach statistical significance in patients with high percentages of circulating PD-L1^+^ monocytes: NR (95% CI NR-NR) vs. 9.13 months (95% CI 6.34 to 11.92), *p* = 0.092 (Supplementary Fig. 2M). High percentages of circulating PD-L1^+^ monocytes without CNS metastases [NR (95% CI NR to NR) vs. 8.42 months (95% CI 5.71 to 11.13), *p* = 0.070 (Supplementary Fig. 2P)], ECOG-PS 1 [NR (95% CI NR to NR) vs. 9.13 months (95% CI 6.87 to 11.38), *p* = 0.051 (Supplementary Fig. 2S)].

No significant differences in median OS were found according to the rest of PD-L1^+^ subpopulations or PD-L1^+^ platelets (Supplementary Fig. 2I-O), nor according to the presence of liver, bone metastases and with ECOG-PS 1 in those cells (Supplementary Table 3).

### Immune-related adverse events (irAE) characteristics

The characteristics of irAEs are summarized in Table 2. Sixteen patients (39%) experienced 25 irAEs of any grade. Median number per patient was 1 [IQR 1–3], with 6 patients (37.5%) experienced multiple events. hypothyroidism (19.5%) and hyperthyroidism (9.8%) were the most frequent irAEs. Regarding severity, 9 irAEs (36%) were G1, 12 irAEs (48%) were G2, 3 irAEs (12%) were G3 and 1 irAE (4%) was G5. Supplementary Table 4 summarizes the patient´s clinical characteristics in those who experienced irAEs and those who did not.

**Table 2:** Description of irAEs

irAES	Patients with irAEs (*N* = 16)			Median time to onset – weeks [range]
	All grade irAES *n* = 25 (%)*	Grade 3–5 irAES *n* = 4 (%)	Requiring corticosteroids (≥10 mg prednisone equivalent) *n* = 10 (%)**	
Hypothyroidism	8 (19.5)	0	2 (4.9)	21.21 [12.71–84.71]
Hyperthyroidism	4 (9.8)	1 (2.4)	2 (4.9)	12.86 [5.71–61.71]
Cutaneous rash	2 (4.9)	0	1 (2.4)	63.43 [55.71–71.14]
Pruritus	2 (4.9)	0	1 (2.4)	30.64 [5.57–55.71]
Diarrhea	2 (4.9)	0	0	15.86 [2.71-29.0]
Renal failure	2 (4.9)	0	1 (2.4)	13.43 [1.86-25.0]
Pneumonitis	2 (4.9)	1 (2.4)	1 (2.4)	43.71 [35.57–51.86]
Neurological syndrome	1 (2.4)	1 (2.4)	0	6.43
Arthralgia	1 (2.4)	1 (2.4)	1 (2.4)	37.71
Hypophysitis	1 (2.4)	0	1 (2.4)	11.86

*Patients could experience > 1 irAE (25 irAEs in 16 patients)

** only patients receiving of ≥10 mg prednisone or equivalent daily for the treatment of immune-related adverse events are reported

*irAE*: immune-related adverse event

Patients with bone metastases developed significantly fewer irAEs (*p* = 0.005), and a trend toward fewer irAES was observed in patients with a BMI < 25, (*p* = 0.06). Those who developed irAEs had non-significantly longer median PFS (7.58 months (95% CI 1.26–13.90) vs. 6.65 months (95% CI 5.32–7.97), *p* = 0.167) and median OS (14.90 months (95% CI 5.54–24.27) vs. 9.13 months (95% CI 7.10-11.15), *p* = 0.082).

### High percentages of baseline PD-L1^+^ circulating neutrophils as predictors of irAEs

**Fig. 4:**
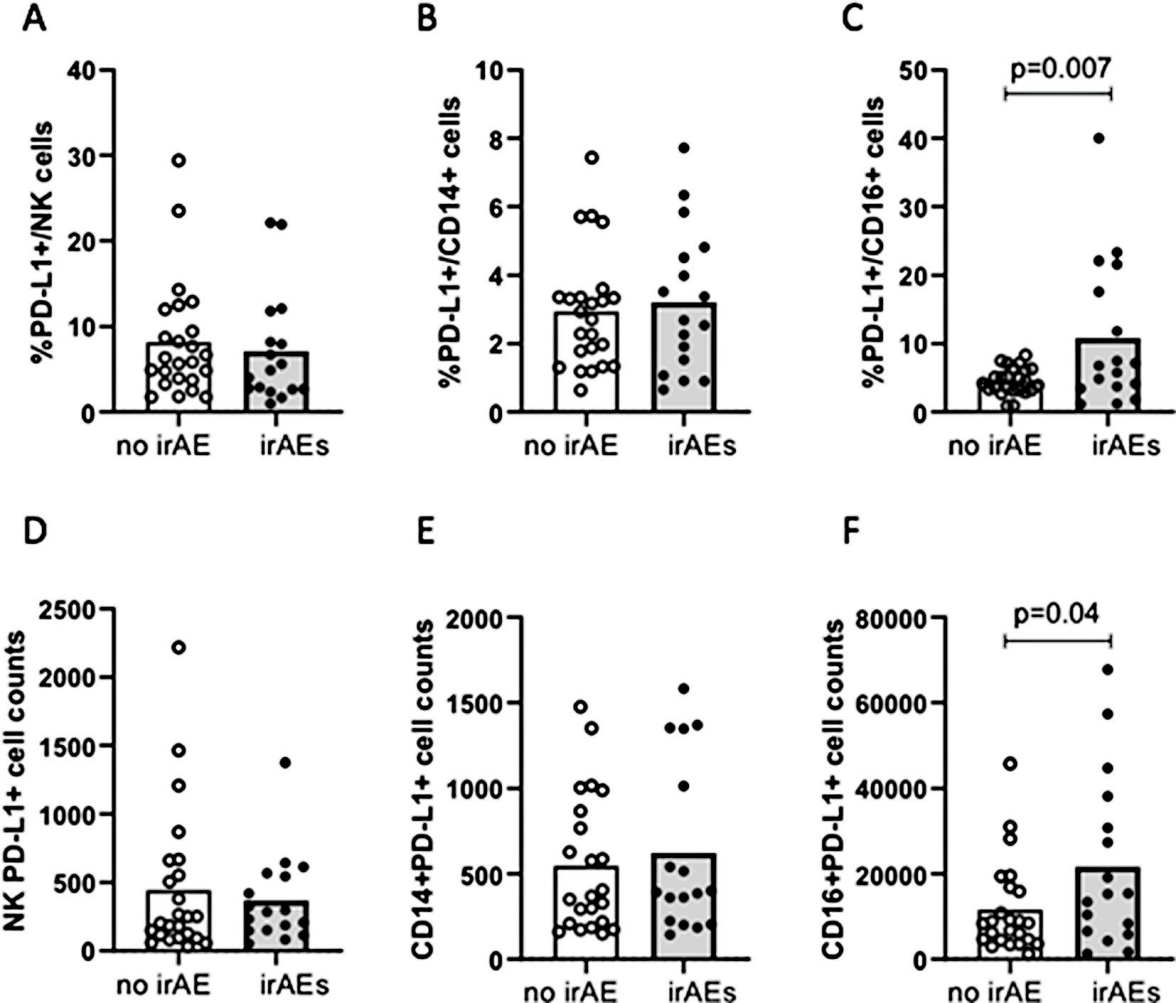
Relation between the presence of irAEs and the percentage of PD-L1^+^ circulating cells in patients with ES-SCLC treated with durvalumab-platinum-etoposide. **A**: percentage of PD-L1^+^ natural killer cells. **B**: percentage of PD-L1^+^ monocytes. **C**: percentage of PD-L1^+^ neutrophils. **D**: count of PD-L1^+^ natural killer cells. **E**: count of PD-L1^+^ monocytes. **F**: count of PD-L1^+^ neutrophils. *irAE*: immune-related adverse event

Patients who developed irAEs presented higher levels of PD-L1^+^ neutrophils at baseline, both for percentages (*p* = 0.007) and for absolut counts (*p* = 0.04) (Fig. 4C and F). Patients with high percentages of PD-L1^+^ neutrophils also developed more non-dermatological irAEs (*p* = 0.013) and higher-grade irAEs (G1–2 vs. G3–5, *p* < 0.001) (Fig. 4). Other PD-L1^+^ populations, including monocytes, NK cells, T/B lymphocytes, and platelets, showed no significant association with irAEs or severity (Fig. 4A, AD, 4B, 4E).

## Discussion

To our knowledge, this is the first study to report PD-L1^+^ circulating immune cells as non-invasive prognostic biomarkers for survival as well as predictive for toxicity in ES-SCLC patients. We found that ES-SCLC patients had higher percentages of circulating PD-L1^+^ cells at baseline compared to HD. High percentages of PD-L1^+^ monocytes correlated with longer PFS, while high percentages of PD-L1^+^ neutrophils predicted irAE occurrence and severity. Importantly, percentages of PD-L1^+^ monocytes subgroups were well-balanced for number and baseline characteristics. OS trends were consistent but did not reach statistical significance, likely due to limited sample size. Larger and independent cohorts are warranted for validation, particularly in patients with high percentages of PD-L1^+^ monocytes.

Currently, PD-L1 expression in tumor does not stratify ES-SCLC patients who would benefit from immunotherapy, as patients benefit from adding atezolizumab regardless of PD-L1 immunohistochemistry status [4, 6]. Other potential biomarkers such as TMB [5], MHC expression [11], transcriptome profiles [12] or specific metastatic sites [6, 8, 9] have been postulated, with inconclusive results, thus highlighting the need for novel biomarkers to guide our treatment decisions.

PD-L1 expression in circulating immune cells has been reported as predictive [28] in other solid tumours [39, 40], especially in monocytes [29]. However, these findings remain controversial, since a negative correlation between high levels of monocytes and shorter survival has also been reported [41, 42]. It is important to underline that PD-L1 expression in immune cells has been described: as PD-L1 expression levels (the amount of PD-L1 expressed by a single cell on their surface) or as the percentage of circulating PD-L1^+^ cells (the number of cells expressing PD-L1 out of the total number of cells). In light of this, this information should be provided when reporting these levels and authors should clarify which defition they are referring to. Our study focused on the percentage of PD-L1^+^ cells as previously performed in NSCLC and using the same cut-off values, rather than surface expression intensity, clarifying methodology for reproducibility [29, 35].

Regarding cut-off values, we statistically pre-specified the 95% CI derived from HD, anchoring the threshold to a biological reference population. This approach reduces risks of overfitting and p-hacking by avoiding data-driven selection and aligns with recommendations for prespecification. Nevertheless, alternative outcome-based methods were considered during study design, including Restricted Mean Survival Time estimation based on each value, Area Under the Curve - AUC - analysis based on 1-year event-free rates (excluding censored data), or median split. These are outcome-based methods that estimate performance rather than biological deviation from normal values and might introduce bias if cut-off values are derived without external validation. Additionally, potential variability across platforms and populations may be important aspects to be considered, as they may impair reproducibility and external validation.

Our results underscore monocytes as key innate immune regulators influencing survival outcomes, accordingly to other results that highlight the innate immune system as influencing immunotherapy outcomes [41, 42]. Conversely, high percentages of PD-L1^+^ platelets did not associate with survival in ES-SCLC, contrasting with previous NSCLC studies, where a significantly longer PFS was observed in patients with high levels of PD-L1^+^ platelets at baseline [43].

Regarding irAEs, patients with high percentages of PD-L1^+^ neutrophils developed a higher rate of irAEs (*p* = 0.003), including non-dermatological irAEs and higher grade irAEs (G1-2 vs. G3-4-5). To our knowledge, this is the first time to ever report this information on ES-SCLC patients. A trend toward better survival was observed in patients who experienced irAEs, as previously reported in other studies [44]. However, immortal time bias arises when irAEs are treated as fixed covariates, as patients must survive long enough to experience an irAE, potentially overestimating survival outcomes for those who develop irAEs. Exclusion of early discontinuation can also introduce posible selection/survival bias, as patients who discontinue treatment early, due to irAEs, may be underrepresented. Landmark analysis and time-dependent Cox models are recommended to mitigate this bias.

Bone metastases and lower BMI were associated with fewer irAEs, consistent with previous NSCLC observations [45–47]. This suggests interactions between immune cell subsets, systemic inflammation, and tumor microenvironment may modulate irAE risk.

Our study has several strengths. This is a sub-analysis of the CANTABRICO trial, which guarantees a homogeneous prospectively collected cohort, standardized methodology, and adequate follow-up. We propose a rapid, economical and non-invasive method for identifying a subgroup of patients with better outcomes.

Despite our relevant findings, our study presents some limitations. These include the modest sample size (although large enough to be statistically analysed), single time-point assessment of PD-L1^+^ cells, and lack of an independent validation cohort. Given the exploratory nature and the sample size, we hypothesize that our findings could support treatment decission if validated in a larger and independent cohort. In addition, we acknowledge the multiple testing and limited power to avoid risk of overinterpretation.

Given that PD-L1 expression is dynamic during treatment and could influence survival outcomes or toxicity, as previously observed in NSCLC patients [22], future studies should explore the longitudinal PD-L1 expression on circulating immune cells dynamic to refine predictive accuracy.

In summary, despite the exploratory nature of our study, PD-L1^+^ monocytes represent a promising non-invasive predictive biomarker for survival, and PD-L1^+^ neutrophils for irAE prediction in ES-SCLC patients treated with first-line durvalumab plus platinum-based chemotherapy, a setting with an urgent need to improve outcomes and where other biomarkers are not helpful to select patients who benefit most across cohorts. These findings might be hypothesis generating for patient stratification and support treatment decision in this high-risk population.

## Supplementary Information

Below is the link to the electronic supplementary material.

Supplementary Material 1

## Data Availability

The datasets used and/or analysed during the current study are available from the corresponding author on reasonable request.
