# Supplementary material for: Baseline PD-L1 expression on circulating immune cells as a predictor of survival and immune-related adverse events in extensive-stage small-cell lung cancer patients treated with durvalumab and carboplatin-etoposide (NCT04712903 Trial)
Source: J Transl Med. 2026 Feb 28;24:471. doi: 10.1186/s12967-026-07896-7 (PMC13059145; doi:10.1186/s12967-026-07896-7)
Supplement: Supplementary file 1 — Supplementary Material 1 [file 12967_2026_7896_MOESM1_ESM.docx]

**Supplementary Table 1.** Distribution of low and high percentages of patients according to each PD-L1^+^ subpopulation.

| **Category – N (%)** | **Low % group – N (%)** | **High % group – N (%)** |
| --- | --- | --- |
| PD-L1^+^CD4^+^ LT | 27 (65.9) | 14 (34.1) |
| PD-L1^+^CD8^+^ LT | 24 (58.5) | 17 (41.5) |
| PD-L1^+^CD20^+^ LB | 22 (53.7) | 19 (46.3) |
| PD-L1^+^ NK cells | 31 (75.6) | 10 (24.4) |
| PD-L1^+^neutrophils | 36 (87.8) | 5 (12.2) |
| PD-L1^+^monocytes | 24 (58.5) | 17 (41.5) |
| PD-L1^+^platelets^+^ | 27 (65.9) | 13 (31.7) |

*Data from 1 patient IS missing due to laboratory issue.

*LT*: lymphocytes T*; NK:* natural killer**.**

**Supplementary Table 2**. Patient characteristics and outcomes according to percentages of PD-L1^+^ monocytes.

| **Patients** | **Low % of PD-L1**^+^ **monocytes** | **High % of PD-L1**^+^ **monocytes** | ***p-value*** |
| --- | --- | --- | --- |
| **Category – N (%)** | **N=24 (90.24)** | **N=17 (68.29)** |  |
| **Follow-up (months)** | **9.34** | **10.14** |  |
| **Sex** |  |  | 0.061 |
| Male | 13 (54.2) | 14 (82.4) |  |
| Female | 11 (45.8) | 3 (17.6) |  |
| **Race** |  |  | 0.152 |
| White | 23 (95.8) | 14 (82.4) |  |
| Others | 1 (4.2) | 3 (17.6) |  |
| **Age – years** |  |  | *0.227 |
| Median | 64 | 66 |  |
| Range | 51-84 | 48-84 |  |
| **ECOG-PS** |  |  | 0.688 |
| 0 | 7 (29.2) | 4 (23.5) |  |
| 1 | 17 (70.8) | 13 (76.5) |  |
| **Smoking habits** |  |  | 0.279 |
| Former smoker | 10 (41.7) | 58.8 (10) |  |
| Current smoker | 14 (58.3) | 7 (41.2) |  |
| **BMI (kg/m2)** |  |  | 0.061 |
| <18,5 | 1 (4.2) | 0 |  |
| 18,5-24,9 | 10 (41.7) | 3 (17.6) |  |
| ≥25 | 13 (54.2) | 14 (82.4) |  |
| Median | 25.6 | 27.7 |  |
| **CNS metastases** | 2 (8.3) | 2 (11.8) | 0.715 |
| **Bone metastases** | 9 (37.5) | 4 (23.5) | 0.344 |
| **Liver metastases** | 7(29.2) | 6 (35.3) | 0.678 |
| **Patients completing 4 cycles of CT-IO** | 22 (91.7) | 15 (88.3) | 0.715 |
| **treatment discontinuation for PD** | 13 (54.2) | 11 (64.7) | 0.500 |
| **Death reason: PD** | 16 (66.7) | 8 (72.7) | 0.449 |
| **Patients alive at data cut-off** | 5 (20.8) | 6 (35.3) | 0.303 |
| **Patients with irAEs** | 8 (33.3) | 8 (47.1) | 0.375 |
| **Median PFS (months) (95% CI)** | 5.97 (4.65-7.28) | 8.97 (NR-NR) | **0.007 |
| **Median OS (months) (95% CI)** | 9.13 (6.34-11.92) | NR (NR-NR) | **0.092 |

*(All Chi-square, but *U Mann-Whitney, **Log-rank test)*

*CNS*: central nervous system*; ECOG-PS:* Eastern Cooperative Oncology Groups performance status*; BMI*: Body Mass Index; *PFS:* progression-free survival; *OS*: overall survival; *CT-IO:* chemo-immunotherapy*; PD:* progressive disease*; irAEs*: immune-related adverse events.

**Supplementary Table 3.** Survival outcomes according to percentages of PD-L1^+^ CD4+/CD8+/CD20+/CD3-neutrophils/neutrophils/PD-L1^+^ PLT+ in patients without CNS metastases/bone metastases/liver metastases and in patients with ECOG-PS 1.

| **Patients** | **N** | **PFS -months (95% CI)** | **p-value** | **OS – months (95% CI)** | **p-value** |
| --- | --- | --- | --- | --- | --- |
| **without CNS M1** | 37 |  |  |  |  |
| PD-L1^+^ CD4^+^ LT low | 25 | 6.806 (6.175-7.438) | 0.710 | 11.032 (9.611-12.454) | 0.290 |
| PD-L1^+^ CD4^+^ LT high | 12 | 6.258 (5.108-7.408) |  | 7.097 (2.846-11.348) |  |
| PD-L1^+^ CD8^+^ LT low | 22 | 6.774 (5.143-8.405) | 0.951 | 10.194 (7.191-13.196) | 0.700 |
| PD-L1^+^ CD8^+^ LT high | 15 | 6.645 (5.953-7.337) |  | 13.968 (5.979-21.956) |  |
| PD-L1^+^ CD20^+^ LB low | 19 | 5.613 (4.650-6.576) | 0.134 | 9.548 (6.200-12.897) | 0.415 |
| PD-L1^+^ CD20^+^ LB high | 18 | 7.032 (6.026-8.038) |  | 11.032 (5.177-16.888) |  |
| PD-L1^+^ NK cells low | 29 | 6.645 (5.965-7.326) | 0.160 | 10.194 (8.946-11.441) | 0.275 |
| PD-L1^+^ NK cells high | 8 | 8.968 (NR-NR) |  | NR (NR-NR) |  |
| PD-L1^+^ neutrophils low | 33 | 6.774 (5.721-7.827) | 0.362 | 10.194 (9.219-11.169) | 0.586 |
| PD-L1^+^ neutrophils high | 4 | 6.387 (NR-NR) |  | 11.032 (NR-NR) |  |
| PD-L1^+^ platelet^+^ low | 23 | 6.806 (5.645-7.968) | 0.904 | 11.032 (8.760-13.304) | 0.806 |
| PD-L1^+^ platelet^+^ high | 13 | 6.645 (5.547-7.744) |  | 9.548 (6.745-12.352) |  |
| **without bone M1 (N=28)** | 28 |  |  |  |  |
| PD-L1^+^ CD4^+^ LT low | 16 | 10.258 (2.671-17.845) | 0.336 | NR (NR-NR) | 0.033 |
| PD-L1^+^ CD4^+^ LT high | 12 | 6.645 (5.550-7.740) |  | 6.903 (6.520-7.287) |  |
| PD-L1^+^ CD8^+^ LT low | 14 | 10.258 (3.161-17.355) | 0.411 | NR (NR-NR) | 0.557 |
| PD-L1^+^ CD8^+^ LT high | 14 | 6.645 (5.975-7.315) |  | 13.968 (NR-NR) |  |
| PD-L1^+^ CD20^+^ LT low | 13 | 7.581 (3.338-11.823) | 0.441 | NR (NR-NR) | 0.838 |
| PD-L1^+^ CD20^+^ LT high | 15 | 7.387 (NR-NR) |  | NR (NR-NR) |  |
| PD-L1^+^ NK cells low | 21 | 7.032 (6.164-7.900) | 0.112 | 13.968 (NR-NR) | 0.380 |
| PD-L1^+^ NK cells high | 7 | NR (NR-NR) |  | NR (NR-NR) |  |
| PD-L1^+^ neutrophils low | 24 | 7.387 (3.360-11.414) | 0.816 | NR (NR-NR) | 0.675 |
| PD-L1^+^ neutrophils high | 4 | 6.258 (NR-NR) |  | 6.613 (NR-NR) |  |
| PD-L1^+^ platelet^+^ low | 19 | 7.581 (2.673-12.489) | 0.794 | NR (NR-NR) | 0.827 |
| PD-L1^+^ platelet^+^ high | 9 | 7.032 (6.655-7.409) |  | NR (NR-NR) |  |
| **Patients without liver M1** | 28 |  |  |  |  |
| PD-L1^+^ CD4^+^ LT low | 19 | 6.806 (5.476-8.137) | 0.985 | NR (NR-NR) | 0.333 |
| PD-L1^+^ CD4^+^ LT high | 9 | 6.903 (6.149-7.657) |  | 7.097 (6.531-7.662) |  |
| PD-L1^+^ CD8^+^ LT low | 17 | 6.903 (5.298-8.508) | 0.776 | 11.032 (9.080-12.984) | 0.519 |
| PD-L1^+^ CD8^+^ LT high | 11 | 6.806 (5.893-7.720) |  | NR (NR-NR) |  |
| PD-L1^+^ CD20^+^ LB low | 16 | 6.000 (3.977-8.023) | 0.156 | 10.194 (6.400-13.987) | 0.502 |
| PD-L1^+^ CD20^+^ LB high | 12 | 7.387 (5.744-9.030) |  | 13.968 (NR-NR) |  |
| PD-L1^+^ NK cells low | 24 | * |  | * |  |
| PD-L1^+^ NK cells high | 4 | * |  | * |  |
| PD-L1^+^ neutrophils low | 24 | 6.806 (5.916-7.697) | 0.527 | 11.065 (NR-NR) | 0.789 |
| PD-L1^+^ neutrophils high | 4 | 6.387 (NR-NR) |  | 11.032 (NR-NR) |  |
| PD-L1^+^ platelet^+^ low | 21 | 7.387 (6.181-8.593) | 0.573 | 13.968 (NR-NR) | 0.511 |
| PD-L1^+^ platelet^+^ high | 7 | 6.645 (5.652-7.639) |  | 6.903 (6.158-7.648) |  |
| **Patients with ECOG-PS 1** | 30 |  |  |  |  |
| PD-L1^+^ CD4^+^ LT low | 19 | 6.806 (5.889-7.724) | 0.904 | 10.258 (8.240-12.276) | 0.358 |
| PD-L1^+^ CD4^+^ LT high | 11 | 6.645 (5.636-7.654) |  | 9.548 (5.560-13.537) |  |
| PD-L1^+^ CD8^+^ LT low | 17 | 6.774 (5.560-7.989) | 0.604 | 10.194 (8.676-11.712) | 0.801 |
| PD-L1^+^ CD8^+^ LT high | 13 | 6.806 (5.481-8.132) |  | 13.968 (6.608-21.327) |  |
| PD-L1^+^ CD20^+^ LB low | 13 | 5.968 (5.134-6.801) | 0.240 | 9.548 (6.821-12.276) | 0.711 |
| PD-L1^+^ CD20^+^ LB high | 17 | 7.032 (6.252-7.813) |  | 10.258 (7.476-13.040) |  |
| PD-L1^+^ NK cells low | 22 | 6.645 (6.015-7.275) | 0.147 | 9.613 (8.315-10.910) | 0.286 |
| PD-L1^+^ NK cells high | 8 | 8.968 (NR-NR) |  | NR (NR-NR) |  |
| PD-L1^+^ neutrophils low | 26 | 6.774 (5.646-7.903) | 0.353 | 9.613 (8.565-10.660) | 0.576 |
| PD-L1^+^ neutrophils high | 4 | 6.387 (NR-NR) |  |  |  |
| PD-L1^+^ platelet^+^ low | 18 | 6.806 (5.532-8.081) | 0.547 | 11.032 (3.186-18.878) | 0.179 |
| PD-L1+ platelet^+^ high | 11 | 6.258 (4.413-8.103) |  | - 1. .565-11.273) |  |

** Not calculated because all 4 cases were censored due to event not occurring.*

*LT*: lymphocytes T*; NK:* natural killer*; mOS*: median overall survival; *mPFS*: median progression-free survival; *CNS*: central nervous system; *M1*: metastases; *ECOG*: Eastern Cooperative Oncology Groups; *NR*: not reached

**Suplemmentary Table 4.** Patient characteristics according to the presence of irAEs.

| **Category – N (%)** | **irAEs (N=16) (39.0%)** | **No irAEs (N=25) (61.0%)** | ***p-value*** |
| --- | --- | --- | --- |
| **Sex** |  |  |  |
| Male | 10 (62.5) | 17 (68.0) | 0.717 |
| Female | 6 (37.5) | 8 (32.0) |  |
| **Age - median [range]** | 63.5 (52-83) | 65 (48-84) | *0.639 |
| **ECOG PS** |  |  |  |
| 0 | 6 (37.5) | 5 (20.0) | 0.217 |
| 1 | 10 (62.5) | 20 (80.0) |  |
| **BMI (kg/m2)** |  |  |  |
| <18,5 | 0 | 1 (4.0) | 0.062 |
| 18,5-24,9 | 2 (12.5) | 11 (44.0) |  |
| ≥25 | 14 (87.5) | 13 (52.0) |  |
| **CNS metastases** |  |  |  |
| No | 13 (81.3) | 24 (96.0) | 0.120 |
| Yes | 3 (18.8) | 1 (4.0) |  |
| **Bone metastases** |  |  |  |
| No | 15 (93.8) | 13 (52.0) | 0.005 |
| Yes | 1 (6.3) | 12 (48.0) |  |
| **Liver metastases** |  |  |  |
| No | 13 (81.3) | 15 (60.0) | 0.154 |
| Yes | 3 (18.8) | 10 (40.0) |  |

*(All Chi-square but *U Mann-Whitney)*

*irAEs:* immune-related adverse events, *ECOG-PS* Eastern Cooperative Oncology Group- performance status, *BMI*: body mass index, *CNS*: central nervous system.

**Supplementary Figure 1.** Correlation between pretreatment percentages of PD-L1^+^ and survival outcomes (Spearman correlation (R)) in patients with ES-SCLC treated with durvalumab-platinum-etoposide

**A**: PFS and PD-L1^+^CD4^+^ lymphocytes T; **B:** PFS and PD-L1^+^CD8^+^ lymphocytes T; **C:** PFS and PD-L1^+^CD20^+^ lymphocytes B; **D**: OS and PD-L1^+^CD4^+^ lymphocytes T; **E:** OS and PD-L1^+^CD8^+^ lymphocytes T; **F:** OS and PD-L1^+^CD20^+^ lymphocytes B.

B

A

**
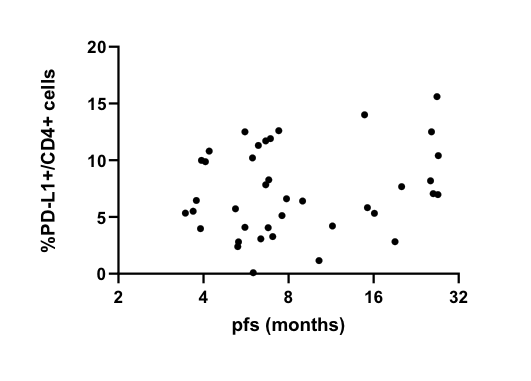

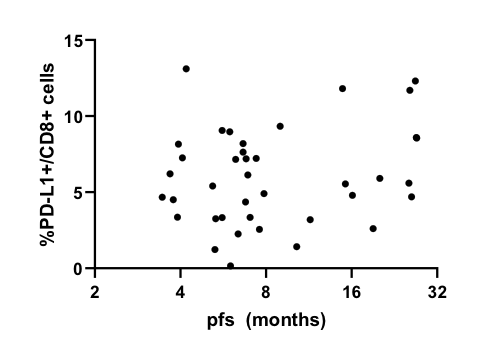
**

D

C

­
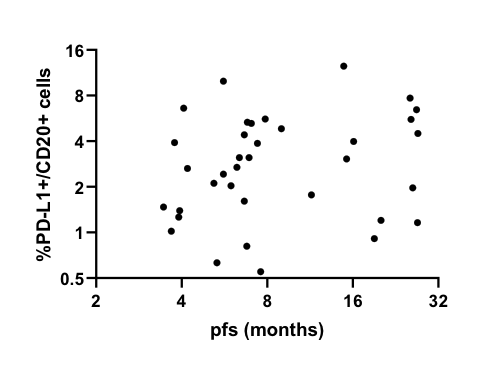

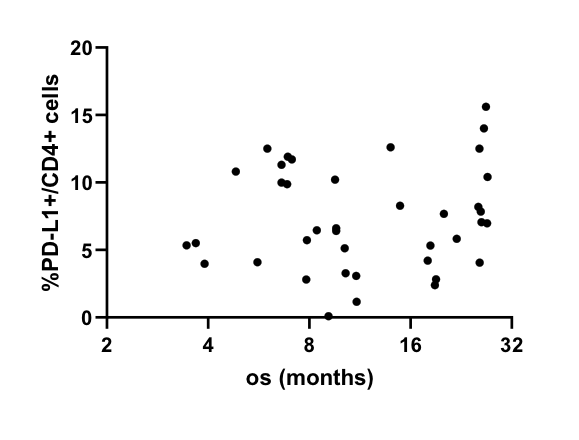


F

E


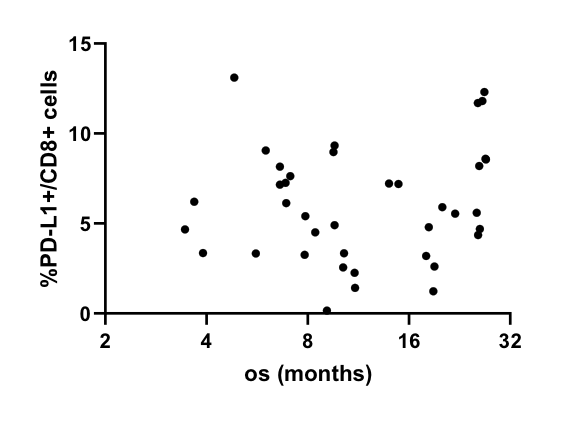

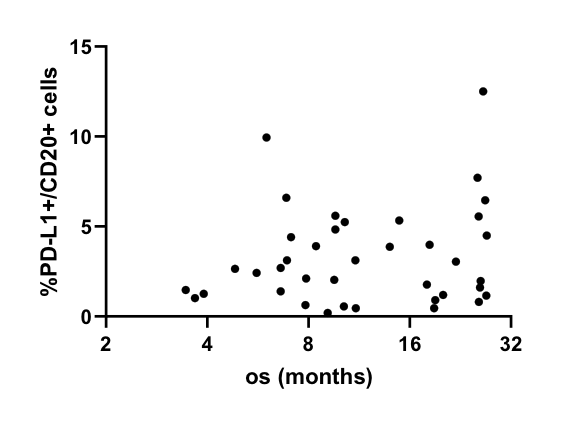


**Supplementary Figure 2.** Kaplan-Meier curves for PFS according to low/high percentages of PD-L1^+^ circulating immune cells **A**: PD-L1^+^CD4^+^ lymphocytes T. **B**: PD-L1^+^CD8^+^ lymphocytes T. **C**: PD-L1^+^CD20^+^ B lymphocytes. **D**: PD-L1^+^ platelet^+^; **E**: PD-L1^+^monocytes in patients without CNS metastases; **F**: PD-L1^+^monocytes in patients without bone metastases; **G**: PD-L1^+^monocytes in patients without liver metastases and **H**: PD-L1^+^monocytes in patients with ECOG-PS 1. Kaplan-Meier curves for OS according to low/high percentages of **I**: PD-L1^+^CD4^+^ lymphocytes T; **J**: PD-L1^+^CD8^+^ lymphocytes T; **K**: PD-L1^+^CD20^+^ B lymphocytes; **L**: PD-L1^+^ NK cells; **M**: PD-L1^+^ monocytes; **N**: PD-L1^+^ neutrophils; **O**: PD-L1^+^ platelet^+^; **P**: PD-L1^+^ monocytes in patients without CNS metastases; **Q**: PD-L1^+^ monocytes in patients without bone metastases; **R**: PD-L1^+^ monocytes in patients without liver metastases and **S**: PD-L1^+^ monocytes in patients with ECOG-PS 1.

A

**
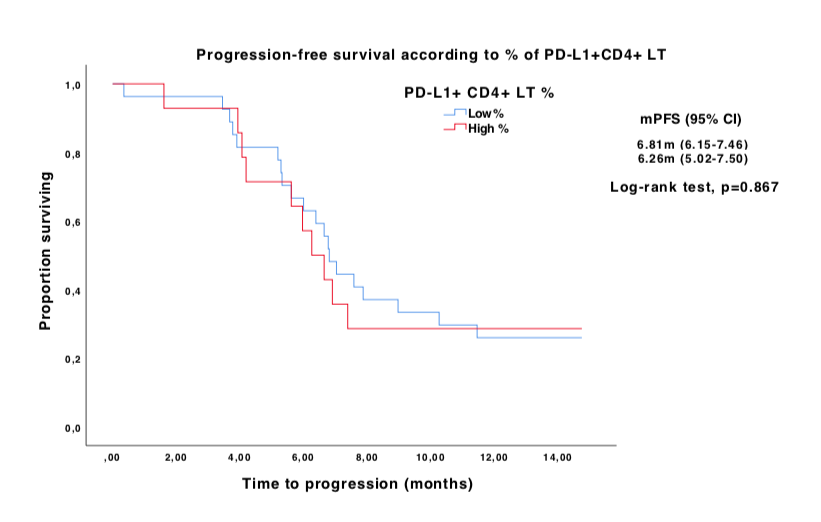
**

B

**
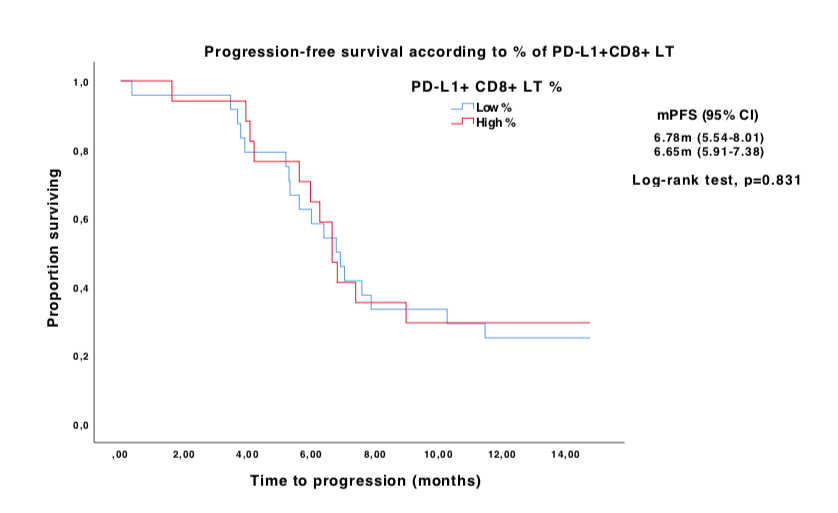
**

C
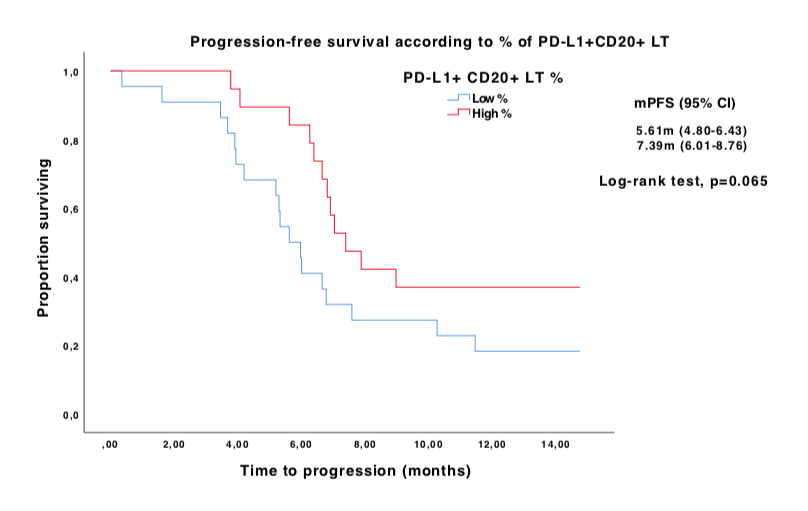


D


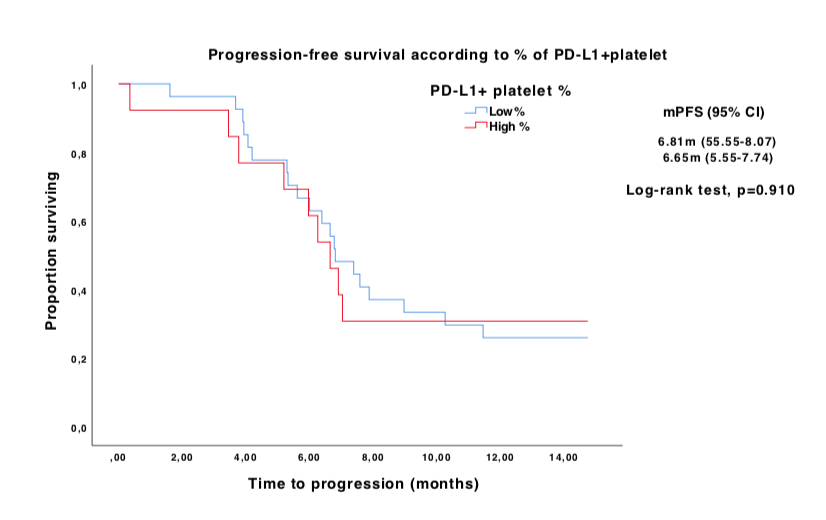


E


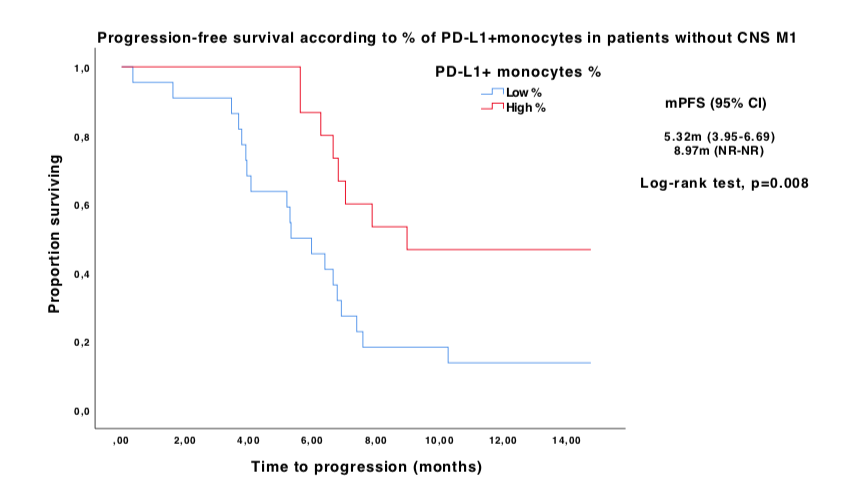


F


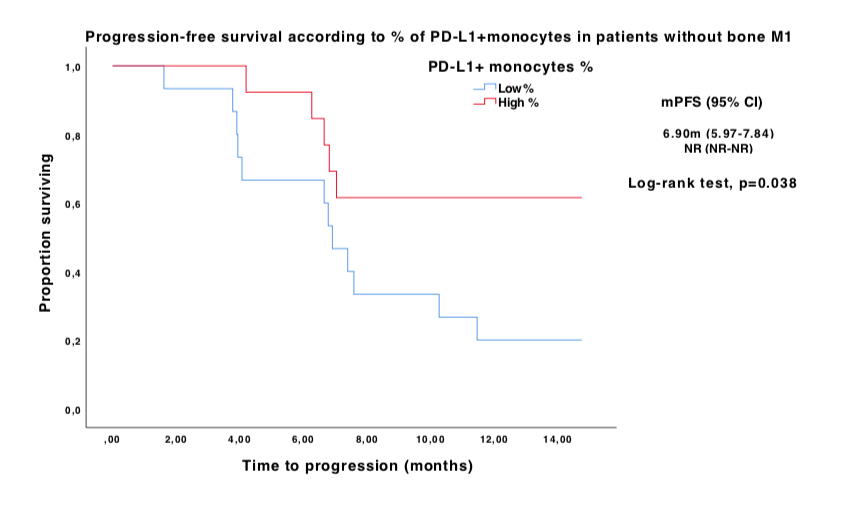


G


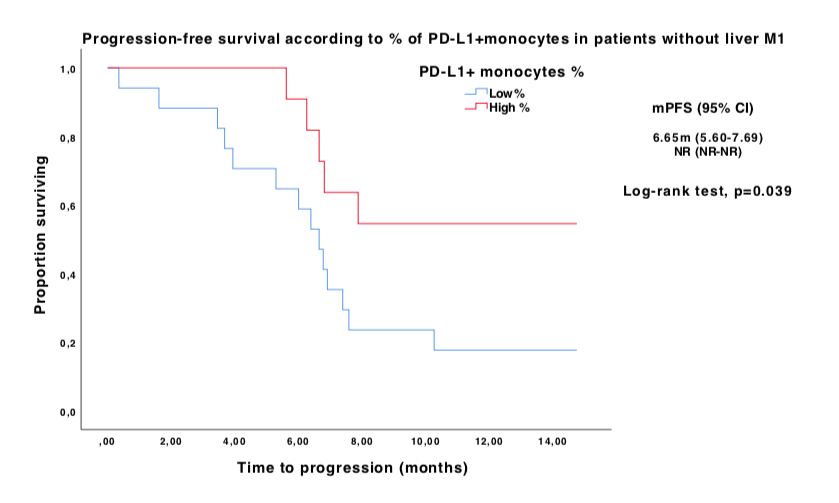


H

**
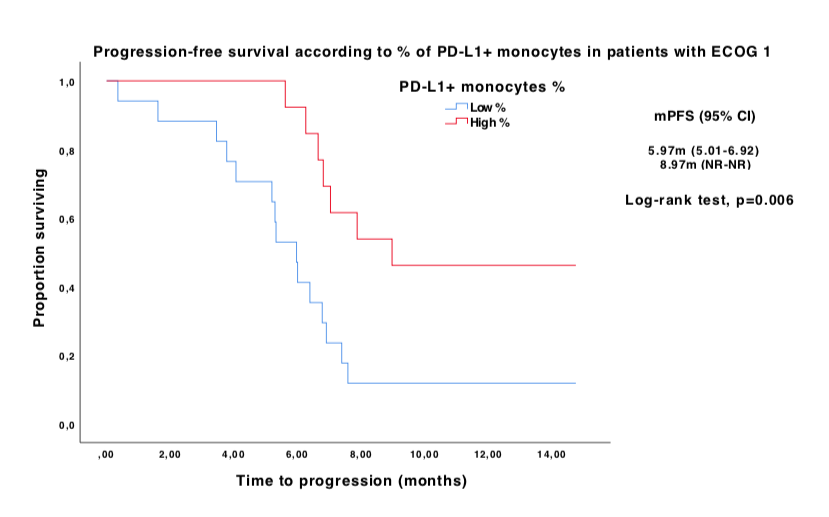
**

I


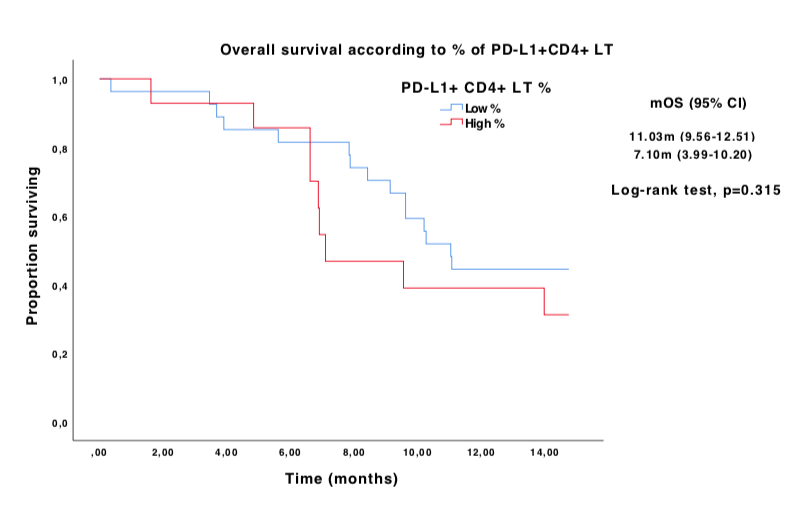


J


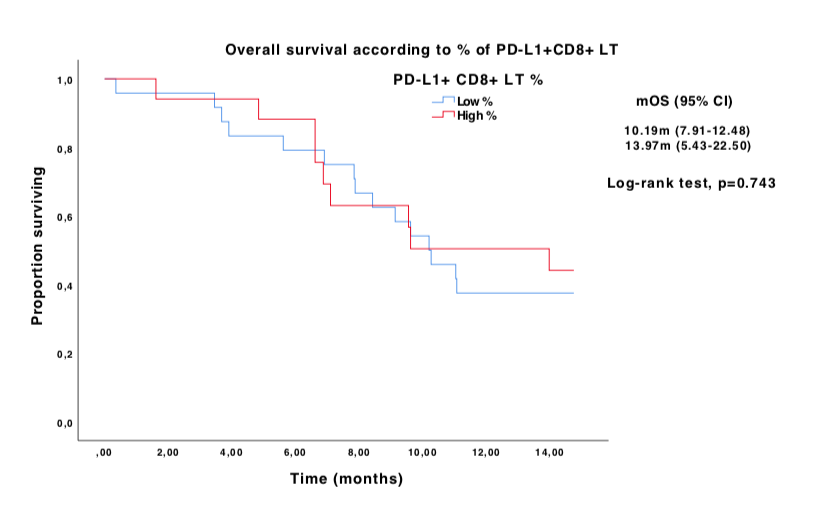


K


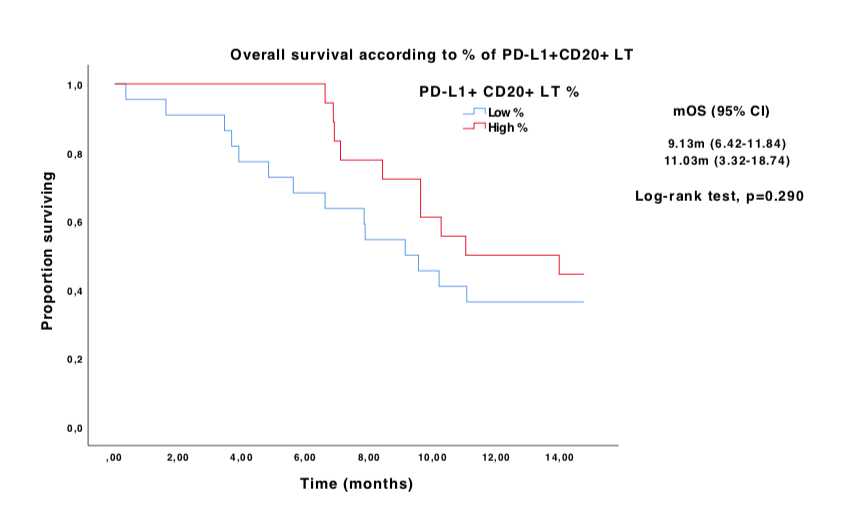


L


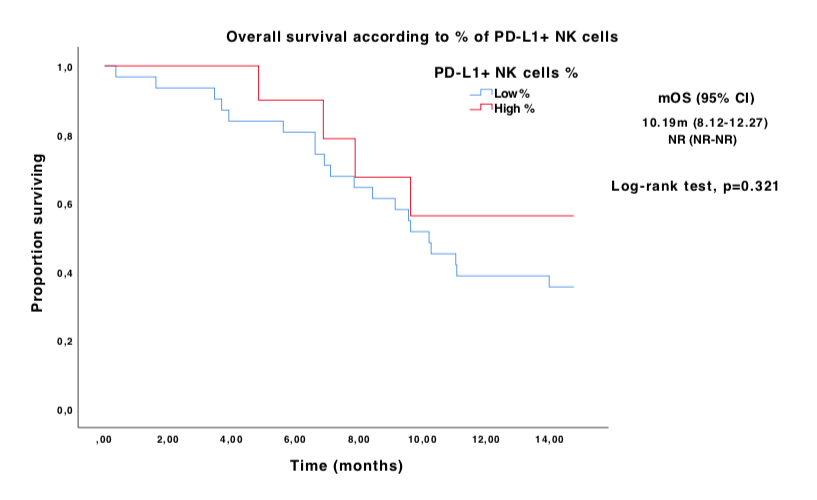


M


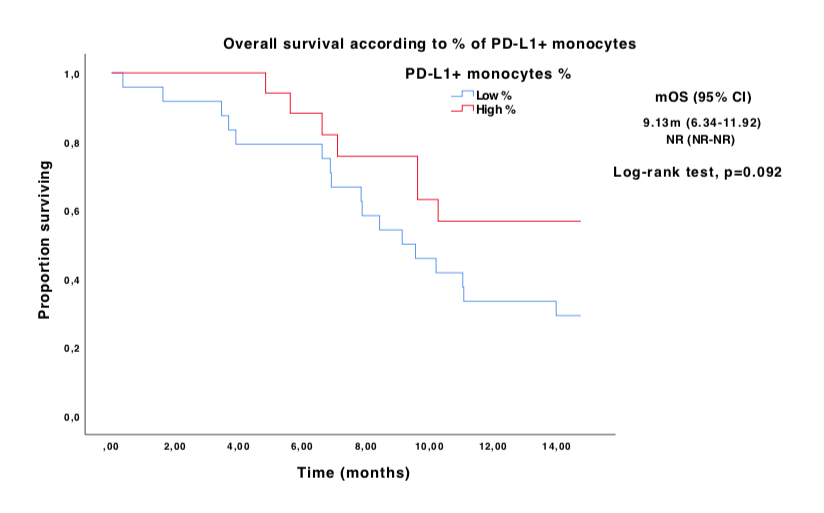


N


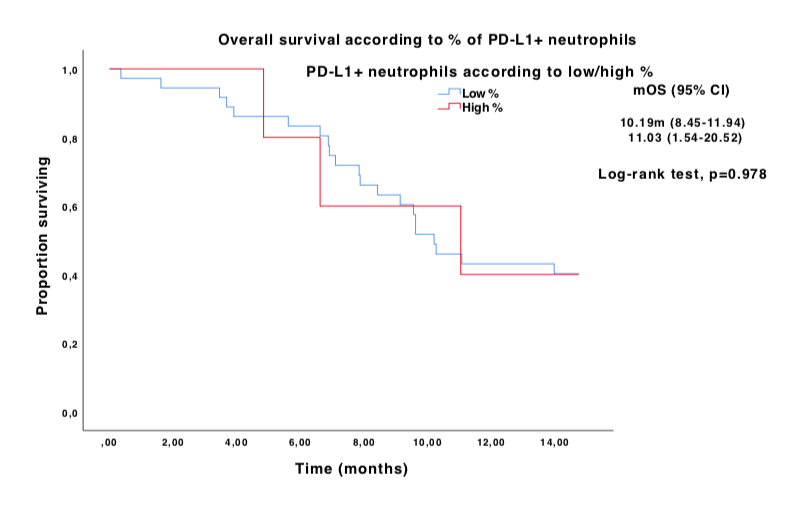


O


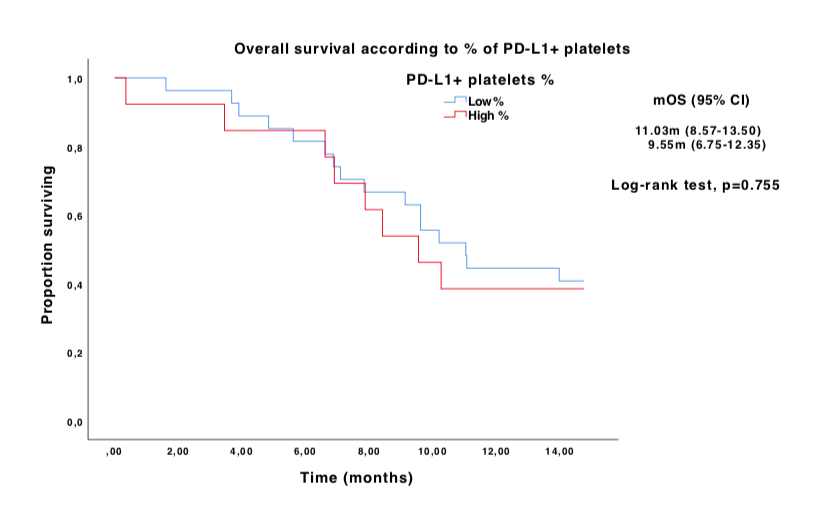


P


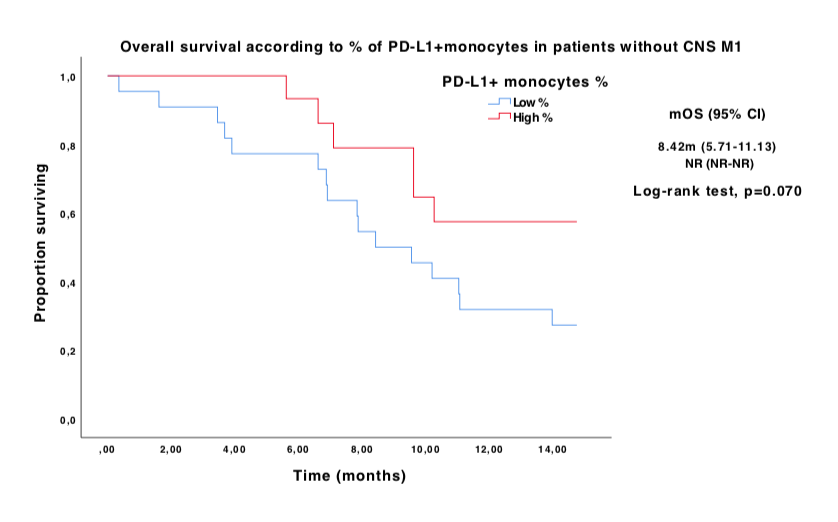


Q

**
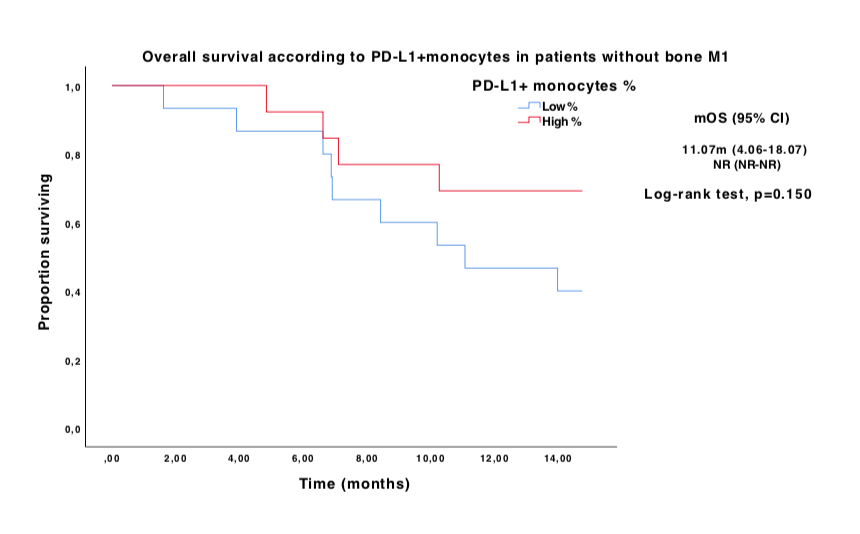
**

R

**
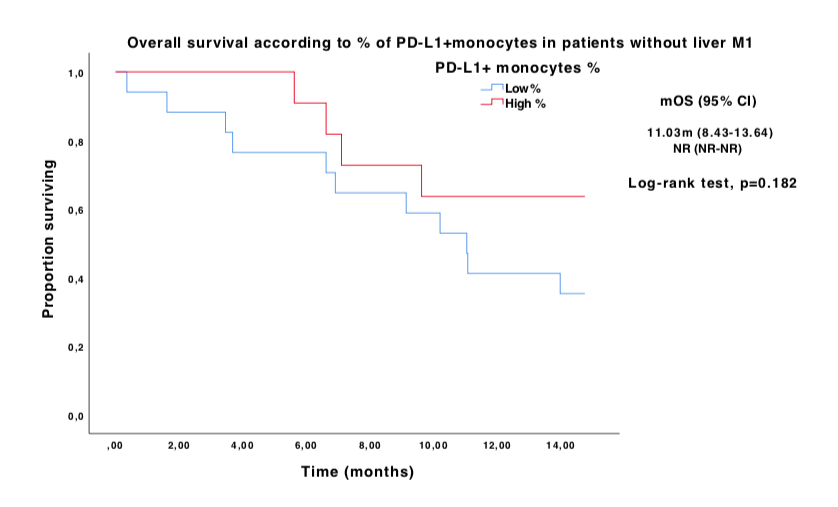
**

S

*
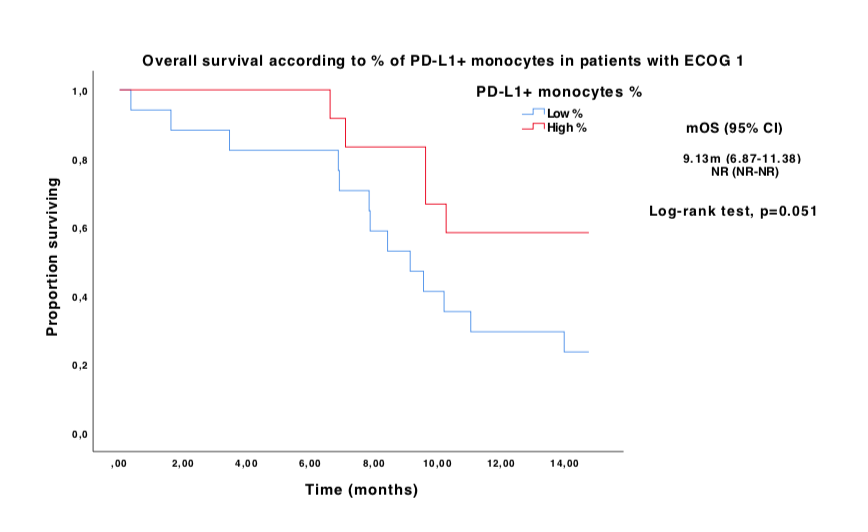
*

*LT:* T lymphocyte*; mOS*: median overall survival; *mPFS*: median progression-free survival; *CNS*: central nervous system; *M1*: metastases; *ECOG*: Eastern Cooperative Oncology Groups

**Supplementary Figure 3.** A model of a blood vessel with sources of PD-L1 in circulating immune cells. Figure created with Biorender.com.

**
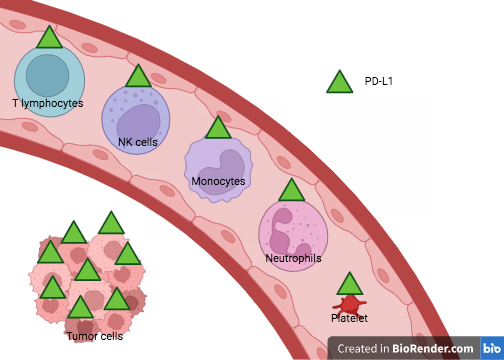
**
